# Male Perspectives on Incorporating Men into Antenatal HIV Counseling and Testing

**DOI:** 10.1371/journal.pone.0007602

**Published:** 2009-11-02

**Authors:** David A. Katz, James N. Kiarie, Grace C. John-Stewart, Barbra A. Richardson, Francis N. John, Carey Farquhar

**Affiliations:** 1 Department of Epidemiology, University of Washington, Seattle, Washington, United States of America; 2 Department of Medicine, University of Washington, Seattle, Washington, United States of America; 3 Department of Global Health, University of Washington, Seattle, Washington, United States of America; 4 Department of Biostatistics, University of Washington, Seattle, Washington, United States of America; 5 Department of Obstetrics/Gynaecology, University of Nairobi, Nairobi, Kenya; 6 Division of Public Health Sciences, Fred Hutchinson Cancer Research Cancer, Seattle, Washington, United States of America; University of Cape Town, South Africa

## Abstract

**Background:**

Male partner involvement in antenatal voluntary HIV counseling and testing (VCT) has been shown to increase uptake of interventions to reduce the risk of HIV transmission in resource-limited settings. We aimed to identify methods for increasing male involvement in antenatal VCT and determine male correlates of accepting couple counseling in these settings.

**Methodology/Principal Findings:**

We invited women presenting to a Nairobi antenatal clinic to return with their male partners for individual or couples VCT. Male attitudes towards VCT and correlates of accompanying female partners to antenatal clinic and receiving couple counseling were determined. Of 1,993 women who invited their partner, 313 (16%) returned with their partners to ANC. Men attending antenatal clinic were married (>99%), employed (98%), and unlikely to report prior HIV testing (14%). Wanting an HIV test (87%) or health information (11%) were the most commonly cited reasons for attending. Most (95%) men who came to antenatal clinic accepted HIV testing and 39% elected to receive counseling as a couple. Men who received counseling with partners were younger, had fewer children, and were less knowledgeable about prevention of mother-to-child HIV transmission (PMTCT) than those who received counseling individually (p<0.05). Only 27% of men stated they would prefer HIV testing at a site other than the ANC. There was agreement between male and female reports for sociodemographic characteristics; however, men were more likely to report HIV preventive behaviors and health communication within the partnership than their partners (p<0.05).

**Conclusions/Significance:**

Offering VCT services to men at antenatal clinic with options for couple and individual counseling is an important opportunity and acceptable strategy for increasing male involvement in PMTCT and promoting male HIV testing.

## Introduction

Mother-to-child HIV transmission (MTCT) remains a significant problem in the developing world despite the development and growing availability of effective prevention methods appropriate for resource-limited settings. Voluntary HIV counseling and testing (VCT) in the context of antenatal care serves as the entry point for targeted prevention of MTCT, and although a majority of pregnant women accept antenatal VCT in these settings [1]–[5], many do not learn their HIV serostatus, take part in prevention programs, or implement interventions to reduce the risk of vertical HIV transmission [4]–[11].

Male partners play a role not only in women's risk of acquiring HIV but also in uptake of antenatal VCT and MTCT prevention programs [12]–[15]. In a previous study, we observed that the involvement of male partners in antenatal VCT was associated with increased uptake of interventions to prevent vertical and sexual HIV transmission [3]. Couple VCT was shown to have greater benefits than accompanying the female partner for individual VCT [3], and further analyses found that couple counseling was similar in terms of cost-effectiveness for preventing MTCT to standard antenatal VCT [16]. Unfortunately, few men accompany their partners to antenatal clinics and even fewer participate in couple counseling when it is available [3], [17].

There is limited research about men's involvement in VCT and the antenatal setting in the developing world from the male perspective. Correlates of male HIV testing behavior and men's reasons for obtaining HIV tests have been examined in sub-Saharan Africa using population-based studies and within workplace- and clinic-based VCT trials [18]–[21]. Reporting HIV risk behaviors generally appeared to be associated with increased testing behavior, but results regarding sociodemographic characteristics varied across settings. Men whose female partners are seeking antenatal care may be influenced by different factors when making decisions regarding participation in VCT, including couple or reproductive characteristics. Much of the research on male involvement in antenatal care and MTCT has relied on women's reports regarding their male partners and has addressed male characteristics and behaviors associated with women's participation in MTCT prevention services [2], [15], [22]–[23]. Only one study has directly addressed male partner correlates of participation in couple VCT, and this study found no differences in the educational attainment and employment status of male partners of women who were couple- versus individually-counseled in an antenatal setting [24].

This prospective, two-week study of men presenting with their female partners at a Nairobi antenatal clinic aimed to describe male attitudes towards antenatal VCT and determine male characteristics correlated with accompanying female partners to antenatal clinic for VCT and couple counseling. An understanding of male participation in this study may help to identify novel methods for increasing male involvement in antenatal VCT and improve uptake of interventions to prevent vertical and sexual HIV transmission.

## Methods

Between September 2001 and December 2002, women attending a Nairobi City Council antenatal clinic were enrolled into a study of antenatal couple counseling and encouraged to return with their male partners for VCT, as described elsewhere [3]. Men were enrolled into the study after providing written informed consent at initial presentation to the clinic. VCT was conducted individually or with the female partner (as a couple), depending on both partners' preferences. After pretest counseling, a rapid assay was performed for HIV and results were available within 30 minutes. Couples who received counseling together shared their HIV test results as part of the post-test counseling session, whereas partners who were counseled individually were advised to do so on their own after the session. Men and women were asked to return to the clinic two weeks later for additional post-test counseling and questionnaire administration. Baseline questionnaires, administered prior to and after HIV testing, assessed sociodemographics, HIV/AIDS knowledge, attitudes towards VCT, sexual and reproductive histories, and partner relations. An MTCT transmission knowledge score was created by counting how many of 3 modes of vertical HIV transmission (during pregnancy, during delivery, and through breastfeeding) participants spontaneously mentioned when asked when the AIDS virus can be transmitted from mother to infant. Similarly, an MTCT prevention knowledge was created by counting how many of 6 methods of preventing vertical HIV transmission (medications, not breastfeeding, breastfeeding for a short time, woman caring for self, condoms, and caesarian section) participants spontaneously mentioned when asked how an HIV-infected mother can prevent her baby from getting infected. Questions regarding barriers to male participation differed between male and female questionnaires.

Data were analyzed using Stata statistical software version 9.2 (College Station, Texas, USA). Correlates of male partner attendance at the antenatal clinic for VCT, couple counseling, and male partners returning for follow-up at two weeks were determined using Wilcoxon rank-sum tests for continuous variables and Pearson χ^2^ and Fisher exact tests for categorical variables. Female partner responses were used to determine correlates of male partner attendance at the antenatal clinic for VCT and male partner responses to determine correlates of couple counseling and return for follow-up. Covariates found to be significant (p<0.05) in univariate analyses were examined in multivariate logistic regression models to determine independent predictors of clinic attendance, couple counseling, and return for follow-up. In cases of collinearity, evaluated using Spearman's correlation coefficients and changes in the standard errors of regression coefficients, only the covariate hypothesized to be most representative of the relationship with the outcome was included. Significance in multivariate models was determined using the likelihood ratio test. Male and female partner responses regarding male and couple characteristics were compared using percent agreement, kappa statistics, and McNemar's tests for categorical variables and percent agreement and concordance correlation coefficients for continuous variables.

The study received ethical approval from the Institutional Review Boards of the University of Washington and the University of Nairobi. Written informed consent was obtained from all study participants.

## Results

### Study population

Between September 2001 and December 2002, 3,137 women presented to the Nairobi antenatal clinic and 2,104 (67%) enrolled into the study and accepted HIV testing. Among these 2,104 women, 1,993 (95%) reported informing their male partners of the availability of HIV testing. Of the 1,993 men who were informed of the availability of HIV testing, 313 (16%) accompanied their partners to the antenatal clinic. Among these, 297 (95%) received HIV testing, of whom 31 (10%) were HIV-seropositive. Of 296 couples for which both partners received HIV testing, 114 (39%) were counseled as a couple and 170 (57%) men returned for the follow-up visit at two weeks. Sociodemographics, sexual and reproductive histories, partner relations, and HIV/AIDS knowledge of the cohort are described in Tables 1 and 2. Men who accompanied their partners were aged 19–53 years. Almost all were in monogamous marriages (97%) and lived with their partners (98%), and more than half (55%) had at least one living child.

**Table 1:** Characteristics of men who accompanied their partners to an antenatal clinic for voluntary HIV counseling and testing.

Characteristics	N	Category	Median or No.	IQR or %
*Sociodemographics*				
Age (y)	313		28	26 to 33
Education level	313	None	2	1%
		Primary	119	38%
		Secondary	141	45%
		College	51	16%
Marital status^a^	313	Married–monogamous	303	97%
		Married–polygamous	9	3%
		Single	1	<1%
Lives with partnera,b	313		308	98%
Duration of relationship (y)	312		2	1 to 5
Employment	311	Salaried job	157	50%
		Self-employed	99	32%
		Casual labourer	49	16%
		Unemployed	6	2%
People per room in house	313		2	2 to 4
Owns home	313		9	3%
Monthly rent (US$)	302		24.29	17.14 to 28.57
*Sexual & reproductive characteristics*				
Age at sexual debut (y)	313		17	15 to 19
Lifetime sexual partners	311		4	2 to 6
History of STI	313		127	41%
Prior HIV testing	313		45	14%
Ever used condom	313		149	48%
Currently uses condoms with partner	313		30	10%
Ever used condom with partner	313		63	20%
Has other sexual partners	312		25	8%
Uses condoms with other partners	24		17	71%
Living children	296	0	133	45%
		1	79	27%
		2	47	16%
		3 or more	37	12%
Plans to have more children	313		225	72%

a These characteristics were significantly different (p<0.05) between men who presented to the antenatal clinic for VCT and those who did not, as reported by their female partners.

b This difference between men who did vs. did not present to the antenatal clinic remained significant in multivariate analysis which included marital status, lives with female partner, previously discussed HIV testing with partner, and thinks baby will benefit from his getting HIV tested by protection from HIV infection.

**Table 2:** Knowledge and attitudes regarding HIV and voluntary counseling and testing of male partners prior to pre-test counseling.

Characteristics	N	Median or No.	IQR or %
*Knowledge*			
Knows HIV+ mothers can transmit HIV to their babies	313	310	99%
Knows only some HIV+ mothers transmit HIV to their babies^a^	309	100	32%
MTCT transmission knowledge score (0–3)^b^	310	3	2 to 3
MTCT prevention knowledge score (0–6)^c^	310	3	2 to 4
*Attitudes*			
Previously discussed HIV testing with female partnerd,e	312	188	60%
Thinks offering HIV testing to men and their partners is better than to men alone	313	311	99%
Would prefer HIV testing at another site:	313	84	27%
At special HIV screening site	84	63	75%
At workplace	84	14	17%
At STD clinic	84	5	6%
Accompanied partner to clinic because:			
wanted HIV test	304	263	87%
wanted information	304	34	11%
Thinks some men have not accompanied their partners because:			
they fear knowing they are infected	304	269	89%
they do not want to come to an antenatal clinic	304	11	4%
they are too busy	304	14	5%
Will personally benefit from knowing HIV status^d^:	312	305	98%
By protecting current sexual partners	305	181	59%
By increased knowledge about his health	305	250	82%
By planning for future	305	163	53%
Thinks baby will benefit from his getting HIV tested^f^:	313	303	97%
By protection from HIV infection^d^	303	274	90%
By planning for future	303	65	21%
If tests HIV positive, will confide in female partner	313	286	91%
If tests HIV positive, will change plans for number of children	313	204	65%

a Of those who knew that HIV+ mothers can transmit HIV to their babies.

b Counted how many of 3 modes of vertical HIV transmission (during pregnancy, during delivery, and through breastfeeding) participants spontaneously mentioned.

c Counted how many methods of preventing vertical HIV transmission (medications, not breastfeeding, breastfeeding for a short time, woman caring for self, condoms, and caesarian section) participants spontaneously mentioned.

d These characteristics were significantly different (p<0.05) between men who presented to the antenatal clinic for VCT and those who did not, as reported by their female partners.

e This difference between men who did vs. did not present to the antenatal clinic remained significant in multivariate analysis which included marital status, lives with female partner, previously discussed HIV testing with partner, and thinks baby will benefit from his getting HIV tested by protection from HIV infection.

f Could identify more than one way of benefiting.

### Differences between men who did versus did not present to clinic

Based on female partner reports, the 313 men who presented to the antenatal clinic for VCT were more likely to be in monogamous marriages and live with their partners, as previously reported [3]. They were also more likely to have previously discussed HIV testing with their partner (27% v. 19%, p = 0.001) and be willing to confide in their partner if they tested HIV-seropositive (68% v. 59%, p = 0.004) than men who did not present to the clinic. Living with and reporting having previously discussed HIV testing with female partners remained significantly associated with attending the antenatal clinic in multivariate analysis (OR (95% CI) = 4.34 (1.05 to 18.0) and 1.49 (1.12 to 1.97), respectively). In addition, women whose partners presented to the antenatal clinic were significantly less likely to test HIV-seropositive than women whose partners did not present to clinic (10 v. 16%, respectively; p = 0.015).

### Attitudes towards voluntary counseling & testing

Of the 304 men who completed the post-test questionnaire at the initial visit, 263 (87%) accompanied their partners to the antenatal clinic because they wanted HIV testing and 34 (11%) because they wanted information about HIV or MTCT (Table 2). Most (88.5%) thought that other men had not accompanied their partners because they feared knowing that they were infected, whereas only 11 (4%) thought it was because these men did not want to come to an antenatal clinic, 14 (5%) because they were too busy, and only 1 (<1%) because he thought that his female partner's test was a proxy for his own and therefore saw no need to be tested himself. On the other hand, women whose partners did not present to clinic primarily cited work as the reason men were unable to accompany them to the clinic (84%); a minority of women indicated that their partners did not want to be tested for HIV (7%), were away from Nairobi (4%), or simply refused to accompany them (3%). Almost all (>99%) men thought that VCT should be offered to pregnant women and that offering HIV testing to men and their partners was preferable to offering it to men alone. Only 84 (27%) men attending the antenatal clinic stated they would prefer HIV testing at another site, 63 (75%) of whom would prefer testing at special HIV screening sites and 14 (17%) at their place of work.

### Differences between men who were couple versus individually counseled

Men who participated in counseling with their partners were younger, had fewer children, and were less knowledgeable about modes of MTCT and methods for preventing MTCT than men who received counseling alone (p<0.05, Table 3). They were also more likely to think that they would change their plans regarding the number of future children if they tested HIV-seropositive and less likely to report that their female partners had previously been tested for HIV (p<0.01). They did not, however, differ in HIV serostatus, sexual history, or couple characteristics, such as marital status and duration of relationship. In multivariate analysis, having fewer children, being less knowledgeable about methods for preventing MTCT, and reporting that female partners had not been previously tested for HIV remained significantly associated with couple counseling (p<0.05, Table 3).

**Table 3:** Significant differences^a^ between male partners who received HIV counseling with their partner (couple) versus alone (individual).

		Couple			Individual			Multivariate Model
Characteristics	Category	N	Median or No.	IQR or %	N	Median or No.	IQR or %	Odds Ratio (95% CI)
*Sociodemographics*								
Age (y)^b^		114	27.5	25 to 32	182	29	26 to 33	
*Knowledge prior to pre-test counseling*								
Knows HIV can be transmitted during delivery^b^		113	93	82%	180	166	92%	
MTCT transmission knowledge score (0–3)b,c		113	2	2 to 3	180	3	2 to 3	
MTCT prevention knowledge score (0–6)^d^		113	3	2 to 3^e^	180	3	3 to 4^e^	0.72 (0.55 to 0.95)
*Sexual & reproductive characteristics*								
Living children		108			171			
	0		62	57%^e^		63	37%^e^	Reference
	1		23	21%^e^		50	29%^e^	0.43 (0.23 to 0.83)
	2		10	9%^e^		36	21%^e^	0.28 (0.12 to 0.65)
	3 or more		13	12%^e^		22	13%^e^	0.82 (0.35 to 1.93)
If tests HIV+, will change plans for number of children		114	88	77%^e^	182	109	60%^e^	2.05 (1.06 to 3.93)
Female partner previously tested for HIV		114	12	11%^e^	182	43	24%^e^	0.43 (0.20 to 0.92)

a All characteristics presented in this table are significant at the 0.05 level comparing couple- and individually-counseled men.

b Covariate excluded from multivariate model due to collinearity.

c Counted how many of 3 modes of vertical HIV transmission (during pregnancy, during delivery, and through breastfeeding) participants spontaneously mentioned.

d Counted how many methods of preventing vertical HIV transmission (medications, not breastfeeding, breastfeeding for a short time, woman caring for self, condoms, and caesarian section) participants spontaneously mentioned.

e *p*<0.01.

### Differences between men who returned versus did not return for follow-up

Of the 313 men who accompanied their female partners to the clinic, 171 (55%) returned for follow-up at two weeks. Men who returned for follow-up were not different from those who did not return in terms of sociodemographics, sexual history, couple characteristics, or couple counseling (data not shown). They were, however, more likely to be HIV-seronegative (95% v. 82%, p<0.001), report prior HIV testing (19% v. 8%, p = 0.019), and report having previously discussed HIV testing with their female partner (69% v. 50%, p = 0.001). Also, prior to VCT, they were more likely to think that they would confide in their partner if they tested HIV-seropositive (96% v. 86%, p = 0.002) and that they would be able to protect their baby from HIV infection by getting tested (94% v. 86%, p = 0.019). In multivariate analysis, being HIV-seronegative (OR (95% CI) = 4.62 (1.94 to 11.0)), reporting willingness to confide in female partners if HIV-seropositive (3.83 (1.45 to 10.1)), and thinking that being HIV tested will help protect their babies from HIV infection (2.45 (1.12 to 5.37)) remained significantly associated with return for follow-up (p<0.05).

### Agreement analysis

Female partners were reliable reporters of male partners' age (r = 0.91), education, and employment (percent agreement ≥70%), but were significantly less likely than their partners to report prior male HIV testing and think men would share test results with them if positive (p<0.001, Table 4). Partner reports regarding couple characteristics (marital status, cohabitation, relationship duration, frequency of condom use, socioeconomic status indicators, number of living children) were often in agreement (median percent agreement 87%, IQR 61–88%; r>0.74; Table 4). However, men were significantly more likely than their female partners to report ever condom use and previous discussions regarding HIV testing, family planning, personal health, and finances within this partnership (p<0.02).

**Table 4:** Agreement between male and female partner responses regarding male and couple characteristics.

Characteristics	N	Agreement	Κ or ρ^a^
*Male Characteristics*			
Age (y)^b^	271	-	0.91
Employment	311	70%	0.51
Highest education level	313	73%	0.59
Previously HIV tested	313	82%	0.29^c^
If male tests positive, will confide in female partner	313	67%	0.07^c^
*Couple Characteristics*			
Marital status	312	95%	0.43
Type of marriage	306	61%	0.30
Duration of relationship (y)^b^	312	-	0.82
Lives with partner	313	98%	0.39
Number of living children	280	87%	0.81
Ever used condom with partner	313	81%	0.29^c^
Currently uses condoms with partner	312	88%	0.17
Previously discussed HIV testing with partner	312	52%	0.12^c^
Discuss family planning issues with partner	312	64%	0.23^c^
Discuss personal health issues with partner	312	88%	0.19^c^
Discuss financial issues with partner	313	87%	0.07^c^

a Categorical variables were compared using kappa and continuous variables using the concordance correlation coefficient.

b Only concordance correlation coefficients were assessed for continuous variables.

c These characteristics were significantly more likely to be reported by men than by their female partners using McNemar's test.

## Discussion

This study is one of the first to describe male attitudes towards VCT and correlates of couple VCT in an antenatal setting. One in six men invited by their female partners to the antenatal clinic for VCT attended the clinic. Most accompanied their female partners to the clinic because they wanted an HIV test or information and thought that the primary barrier preventing other men from accompanying their partners was a fear of knowing they were infected. This suggests that there is interest among men regarding antenatal VCT and that offering VCT and health information for men in this setting may hold promise as means for increasing male involvement in VCT and interventions to prevent MTCT. Almost all men who accompanied their female partners thought that they would personally benefit from knowing their HIV status, as would their infants. Increasing the perceived benefits of VCT to both men and their infants through educational or media campaigns may therefore increase male participation in antenatal VCT.

While concerns have been raised that men will not attend antenatal clinics for VCT because men are traditionally not involved in antenatal activities and antenatal services are typically not directed towards men [3], [17], only a quarter of men in this study would prefer VCT at another site, less than 5% thought that other men did not accompany their female partners to the clinic because they did not want to come to an antenatal clinic, and 94% of men rated receiving VCT in the antenatal setting positively. Antenatal clinics, therefore, may be an acceptable location for VCT for men. However, acceptability, motivations, and barriers reported by men attending an antenatal clinic may not be representative of all men with pregnant partners.

Although most men thought that other men did not accompany their partners to the clinic because they feared knowing that they were infected, only a small minority of women whose partners did not accompany them to the clinic thought that fear of HIV testing was the primary barrier to attendance. The majority of these women indicated that work or other time commitments prevented their partners from attending the clinic. Making antenatal services available outside of normal working hours might increase the number of men who are able to accompany their partners to antenatal visits. In our experience, however, adding a Saturday clinic did not have a measurable impact on the proportion of men accompanying their partners to clinic but the clinic was well-attended. It is possible that men use work as an excuse not to attend the clinic for VCT due to their fear of testing and that efforts will need to be made to address this underlying fear in order to increase male HIV testing in any setting.

Men who accompanied their female partners to the clinic appeared to have stronger commitments to their female partners and more open communication regarding HIV within the partnership than men who did not. Similarly, in other studies, being married was correlated with men bringing a partner for VCT [20], and having discussed HIV with one's spouse was associated with both having been HIV tested and being willing to be tested [19]. Greater commitment to a female partner may increase a man's motivation to participate in VCT and in antenatal care, and having discussed HIV in the past may motivate or simplify HIV test-seeking. Encouraging and facilitating discussions regarding HIV/AIDS within relationships through the media and other interventions may increase the number of men accompanying their female partners to antenatal clinics when male VCT is available. Special efforts may be necessary to reach male partners of unmarried women seeking antenatal care.

Differences between men who received couple versus individual VCT may reflect an increased attentiveness among men and women during a first pregnancy that results in greater acceptance of messages promoting the benefits of couple counseling. Similar to previously reported correlates of male HIV testing [18]–[19], receiving couple counseling was associated with being younger and less knowledgeable regarding HIV prevention; however, couple counseling was not associated with HIV risk behaviors as male testing, willingness to be tested, or seeking test results have been in previous studies [18]–[20]. This would suggest that motivation for couple VCT by men in the antenatal setting may have more to do with their partners' pregnancy than their individual risk. Because men used both individual and couple counseling when both partners' preferences influenced how counseling was delivered, antenatal clinics offering services to men should consider including options for both couple and individual counseling. Antenatal clinic staff may wish to spend additional time or develop targeted messages for addressing the benefits of couple counseling with older fathers and their partners in order to increase their rates of participation in couple counseling.

This study had several strengths. It is the first study to address correlates of male participation in couple counseling in the antenatal setting and one of few studies regarding male involvement in VCT and antenatal care in Africa. In addition, we were able to use reports from the men themselves and to compare these with female reports regarding their male partners and shared characteristics. Reasonable agreement existed between male and female responses regarding male and couple sociodemographics, but men were more likely than their female partners to report HIV preventive behaviors and communication about health within the partnership. Although it is preferable to use male reports when studying male involvement in VCT and the antenatal setting, female reports regarding male and couple sociodemographics can be acceptable surrogates. One limitation of our study is that it was conducted in a public antenatal clinic serving an urban population in Kenya and, therefore, may not be applicable to other resource-limited settings, such as rural communities or outside of East Africa. In addition, a number of factors were analyzed, which could result in statistically significant differences between comparison groups that are due to chance alone, although the coherent picture presented by these differences suggests otherwise.

In conclusion, this study suggests that offering VCT services for men at antenatal clinics, with options for both couple and individual counseling, may be an acceptable strategy for increasing male involvement in VCT and PMTCT interventions in resource-limited settings. Additional research will be necessary to guide development of male VCT services in the antenatal setting, and as couple counseling is promoted in resource-limited settings, its incorporation into antenatal clinics should be considered for the distinct benefits it can have in this context for both partners and their children.
